# Medical Journals in New-York

**Published:** 1845-11-01

**Authors:** 


*Medical Journals in New-York.*—Medical Journals appear to be multiplying in our metropolis. In our last No. we acknowled the reception of the first number of the "Intelligencer and Eclectic Gazette," a bi-weekly, devoted to selections from foreign periodicals. We have now to acknowledge the first number of the "New-York Medical and Surgical Reporter," a bi-weekly of 16 pages, at $2,00 per annum, edited by Clarkson T. Collins, M. D., the object of which is to present reports of medical lectures by Professors Mott, Parker, and other teachers connected with the schools and hospitals of that city. The plan strikes us favorably, and if properly carried out, will, we think, prove acceptable to the profession. It has often been a subject of surprise and regret to us, that more effort is not made to develope our own resources, and to cultivate a national, medical literature. The attention of the profession seems to be almost exclusively directed to foreign sources for supplies of medical information. We do not make this remark from any desire to disparage our obligations to other countries, but we sincerely believe that as a profession we do not do justice to ourselves. We have in our larger cities all the resources which can exist any where; our profession contains the necessary talent, energy and industry to render these and other resources available for scientific investigation. Why, then, should we not indulge and encourage a patriotic pride in rendering our domestic resources of medical knowledge second to none in any country! Why should we not have writers and teachers whose names shall carry as much force and authority as those of distinguished personages in France and Britain! Why shall the idea be sanctioned that these or other countries afford facilities for acquiring practical knowledge of diseases which the home student cannot enjoy!—But we have wandered from our subject, viz—the Journals of New-York. Before leaving this subject, we beg leave to intimate to our friend, the editor of the New-York Journal ofMedicine, that we prize his bi-monthly visitation too highly not to feel greatly disappointed when it fails to greet us; and we are selfish enough to remind him that the last number is not in our list of publications received.

